# Lecture on Fever

**Published:** 1856-04

**Authors:** William Stokes


### LECTURE ON FEVER.

BY WILLIAM STOKES, M. D.

I endeavored to convey to you, at our last lecture, that the conditions which have been described under the head of typhoid pneumonia, were probably examples not only of a pathological, but of an anatomical state of parts, different from that which is found in the simple original inflammation of the lung. And it is a great deal easier to say what they are not, than what they are,—to state their negative, rather than their positive characters.

Now I wish to state to you here, that a certain change has occurred in our opinions, as to the origin of the so-called typhoid inflammation of the lung. We at one time held, that it was the co-existence of gastritis or enteritis which gave to the pneumonia the typhoid character. This view was held by us before we had, by that imperceptible power of conviction which arises from experience, admitted the two following principles in their entirety:

1. That symptoms which are diagnostic of local disease, where the patient has not an essential fever, are either altogether valueless, or much lessened in value when such a condition exists; and

2. That the gastric or gastro-enteric lesion is rare, even as a secondary disease in fever; so that when irritation of the structures of the intestinal tubes occurs, it is a remote, tertiary, and accidental phenomenon.

Our present opinion on this matter is, in general, the following: that in cases in which there are, in connection with the signs of typhoid lesion of the lung, evidences of gastro-intestinal disease, both the pulmonary and abdominal lesions spring from the one parent condition, and that so far from the specialties of the pulmonary, being derived from the accidental complication with the abdominal disease, both have a common character originating in the same source. I am quite sure that a large proportion of those cases described as asthenic pneumonia, depending on gastro-intestinal complication, have been examples of essential fever, with the two affections co-existing as secondary lesions.

We have seen that in these cases, I will not say of typhoid pneumonia, but of typhus or typhoid affections of the lung, the various physical signs of pneumonia, singly considered, may be present, and are actually often to be found. They fail, however, very frequently to present themselves in the regular order or succession which is observed in true acute pneumonia.

Now let us inquire whether there is any physical sign peculiar to these cases of typhus pulmonary affections, which does not occur, at least as the rule, in ordinary pneumonia; and I do not know of the existence of any such, unless it be the sign of tympanitic resonance over the diseased lung, a condition first noticed by Dr. Hudson of this city, and to which he attaches some importance. Dr. Hudson states that in certain cases of typhoid consolidation of the lung, the sound on percussion, was very different from that observable in the ordinary condition of hepatization. He describes it as "a tympanitic clearness over the solidified lung, without air being present in the pleura;" indeed he goes so far as to say that in one case the tympanitic clearness on percussion, existed fully to the same degree, and of the same kind as in pneumo-thorax; here the lung was found perfectly solid throughout, with the exceptionof a small extent of the anterior and postero-inferior parts which was still crepitating.

It is very difficult to understand what condition of parts could have caused this singular tympanitic clearness over a solidified lung. When we speak of tympanitic resonance, it must be always borne in mind that the tympanitic sound does not always imply clearness on percussion. When you have a cavity in the centre of a solidified lung, or when you have hepatization of the left lung, in connection with flatulent distension of the stomach, the sound on percussion, though dull as compared with that of the healthy lung, has a distinctly tympanitic character; to this we have long been in the habit of giving the name of tympanitic dullness. I have never found it, however, to simulate the tympanitic resonance which occurs in pneumo-thorax, or in dilatation of the air cells; it is inferior in degree, and different in character. Dr. Hudson met with four cases, in which the observation of this phenomenon was followed by dissection. One was that of a man who died of extensive inflammation of the left lung in the Meath Hospital, in the spring of 1832, in which, at the close of the case, from the hollow sound on percussion at the lower part of the left side, it having been previously quite dull, a pretty general opinion existed that a pneumonic abscess had formed and burst into the pleura. On dissection, the side having been punctured, no air escaped; the lung was red and solid, but without abscess, and the pleura was adherent over two-thirds of its extent. I must, in justice to myself, state that in this case I never entertained the idea that an abscess had opened into the pleura. In another case, the lung was found hard and solid, from chronic pneumonia; and in the two remaining cases, the condition of parts was similar to that which was presented by that first detailed.

Dr. Hudson admits that, in three of those cases, the tympanitic sound might be explained by reference to the distended state of the stomach; but in the fourth, he thinks that the explanation might be found in the faculty with which the vibrations of the air in the bronchus, and its larger divisions, may be supposed to be communicated through a lung in that condition, that is to say, solid throughout; and therefore, not permitting the loss in a mixed medium of solid and healthy lung of such vibrations.

I am quite prepared to admit that, with extensive solidification of the lung, the dull sound, on percussion, may yet have a tympanitic character; but I have seen no case in which this sound could be confounded with that of pneumo-thorax, or of dilatation of the air cells. With reference to the bearings of this question upon the signs of typhoid pneumonia, I can only at this moment remember two cases which are worth detailing to you. In one, tympanitic dullness did occur over the diseased portion of the lung, without our being able to account for it, by any accumulation of air, either in the pleura or in the stomach. The case was of a low putrid character, and I remember suggesting it as a matter just possible, that there might have been a typhoid pneumatosis developed in the diseased lung; but I am sure that we were not able to establish the existence of such a condition on dissection. The case occurred a good many years ago.

In the second case, which was one of manifest typhus fever, the posterior portion of the right lung became solid or nearly so, while the anterior face of the organ preserved its vesicular respiration. Now we found that over this portion of the chest, that is, over the front of the thorax on the right side, the sound as compared with that over the opposite lung, was morbidly clear; it was true tympanitic clearness, not dullness, and it continued for three or four days, and gradually disappeared with the resolution of the posterior solidity: this case occurred in the Hospital last year, and was seen by Dr. Hudson himself. I confess I am quite at a loss to explain the nature or mode of production of this phenomenon. My friend, Dr. Lyons, mentions to me that in a case of asthenic pneumonia, occurring in a patient of intemperate habits, which we saw in consultation, the anterior superior part of the left lung presented, for a couple of days, a condition of morbid clearness, but subsequently became engaged in the general consolidation of the organ.

Dr. Lyons is disposed to regard the abnormal clearness which occurs in these cases, as the result of the increased pressure of the respiratory column of air in the still permeable portions of the pulmonary cells, which he considers, in certain cases, become from this cause, expanded beyond the natural volume. His views are, that the inspired air presses with a certain force on the whole pulmonary surface, and that if a portion of this surface becomes impermeable to air, from solid deposit, occlusion of the tubes which forced it, or other cause, the remaining portion of the pulmonary tissue is acted on by the whole of the inspiratory force, before which it is thus made to expand; this portion of the lung may thus be considered to be in a condition of temporary emphysema, and so gives a correspondingly clear sound on percussion.

There is a circumstance in connection with the resolution of these typhoid or typhus diseases of the lung, different from what is commonly observed in sthenic pneumonia. You know that the true inflammatory hepatization rarely disappears in a sudden manner. It subsides gradually, and the transition-state between dullness and clearness on percussion is generally marked by the "crepitus redux." In the cases before us, however, and especially where the disease is secondary to typhus fever, the resolution, as I have before stated to you, is often singularly rapid, and is often unattended by the crepitus of resolution. If then, you consider the state of solidification simply, we find it on the one hand, to form without the crepitus of the first stage of pneumonia, and on the other, to disappear rapidly, and without the rale of resolution. Thus we are permitted, as it were, to witness the silent and spontaneous development and retrocession of one of the secondary diseases of typhus.

This change from the state of consolidation to that of permeability to air, this rapid change, unattended by the crepitus and resolution, probably shows that the real disease was one unconnected with inflammation, either as a primary or a reactive condition.

You will remember that I suggested to you that some of the cases which have been described as typhoid pneumonia, might be held as examples of an aborted typhus. These were characterized by early consolidation, early disappearance of the typhus state, and a rapid, and often spontaneous subsidence of the local disease. I cannot help thinking that between such cases, and those in which the general disease runs its usual course, there is another class, or category of cases in which the progress of the merely pulmonary disease is marked, more or less, by signs of irritation or inflammation of the lung, which inflammation or irritation is either reactive or specific, or both reactive and specific. And I apprehend that these cases which, as it were, float between the aborted and the perfect typhus, are much more numerous than might be supposed; and in such instances, the case is often treated throughout, without a suspicion of its being really an example of typhus disease having been entertained.

What has now been said, should impress on your minds that rule in practice which I have so often urged upon you, namely, that the rules of diagnosis of local inflammatory disease which are good in ordinary cases, lose their value in a great measure when the patient has typhus fever. This was long ago proved by the researches of Louis on the condition of the brain in fever, and it was the non-recognition of this fact which constituted one of the greatest errors in the system of Broussais. I have told you, that if you gained nothing during the session but the knowledge and full appreciation of this great principle, your time would have been well spent. How many cases have we not had of headache, delirium, watchfulness, or its opposite, coma,—yet without encephalitis; and so it is with the remaining cavities—symptoms of functional alteration are met with in connection with the cerebral, pulmonary, circulating, and digestive systems in fever. They may or may not be attended by organic change, and that organic change, when it does exist, is not necessarily inflammation; and we cannot, I believe, lay down any satisfactory rule of diagnosis which would show, that in one case of local functional disturbance there was organic change, and in another that there was not. But this much we do know, that those groups of symptoms which are diagnostic of local inflammation in a case which is not fever, cease to be so when they occur in a case of typhus. Let this principle be ever present to your minds, for it is impossible to exaggerate its value. Long ago it was acted on empirically by the best physician, who refused to adopt antiphlogistic measures in treating the local symptoms in typhus, and who employed stimulants, irrespective of them, when the general condition seemed to demand such treatment. It now comes before you as the result of an extended and accurate pathological investigation, and the study of the pulmonary phenomena, as we have seen, enables us to go a step further, and to declare that not only are the symptoms of local irritation doubtful or illusive; but that even the physical signs of a pneumonia, when occurring in a case of typhus, are not to be taken as proof that a local inflammation has occurred.

If these things be true so far as our typhus is concerned, it would appear probable, that in other acute diseases, under the influence of a law of periodicity, and, perhaps, in many that arise from the operation of an introduced poison, the same circumstances may be found, so that we might apply to a much larger circle of disease those principles as to the secondary local affections, which appear applicable to typhus fever.—*Virginia Med. & Surg. Journal.*

